# Effects of dietary restriction on adipose mass and biomarkers of healthy aging in human

**DOI:** 10.18632/aging.101122

**Published:** 2016-11-29

**Authors:** Daniele Lettieri-Barbato, Esmeralda Giovannetti, Katia Aquilano

**Affiliations:** ^1^ Department of Biology, University of Rome Tor Vergata, Rome, Italy; ^2^ IRCCS San Raffaele La Pisana, Rome, Italy

**Keywords:** adipose tissue, aging, calorie restriction, fasting, biomarkers, human, longevity

## Abstract

In developing countries the rise of obesity and obesity-related metabolic disorders, such as cardiovascular diseases and type 2 diabetes, reflects the changes in lifestyle habits and wrong dietary choices. Dietary restriction (DR) regimens have been shown to extend health span and lifespan in many animal models including primates. Identifying biomarkers predictive of clinical benefits of treatment is one of the primary goals of precision medicine. To monitor the clinical outcomes of DR interventions in humans, several biomarkers are commonly adopted. However, a validated link between the behaviors of such biomarkers and DR effects is lacking at present time. Through a systematic analysis of human intervention studies, we evaluated the effect size of DR (i.e. calorie restriction, very low calorie diet, intermittent fasting, alternate day fasting) on health-related biomarkers. We found that DR is effective in reducing total and visceral adipose mass and improving inflammatory cytokines profile and adiponectin/leptin ratio. By analysing the levels of canonical biomarkers of healthy aging, we also validated the changes of insulin, IGF-1 and IGFBP-1,2 to monitor DR effects. Collectively, we developed a useful platform to evaluate the human responses to dietary regimens low in calories.

## INTRODUCTION

Aging and wrong lifestyle choices, including inadequate dietary patterns, increase the risk of developing several diseases such as obesity and its-related chronic degenerative diseases. Interestingly, the aging program can be accelerated by obesity [1]. It is thus likely that obesity reduces life- and health span and plays a predominant role in the onset of age-related diseases [2]. In fact, the prevalence of obesity is globally increasing in populations and has become a burden for healthcare systems. Several studies suggest that dietary restriction (DR) regimens (e.g. intermittent fasting, calorie restriction, low calorie diet) reverse obesity and improve health in human by promoting the same molecular and metabolic adaptations that have been shown in animal models of longevity. In particular, DR in humans ameliorates several metabolic and hormonal factors that are implicated in the pathogenesis of an array of age-associated chronic metabolic diseases [3, 4].

At present it is difficult to evaluate the effectiveness of DR on lifespan in humans, so that several works proposed predictive non-invasive biomarkers to evaluate the geroprotective role of DR. However, a miscellaneous of biomarkers is investigated in human intervention studies limiting the statistical robustness of the data. Whether a “biomarker-based” approach could be suitable for evaluating the effectiveness of DR still remains a matter of debate.

Precision medicine is a medical model that proposes the customization of healthcare, with the identification of predictors that can help to find the effectiveness of health-promoting dietary interventions. Biomarkers represent potentially predictive tools for precision medicine but, although affordable 'omics'-based technology has enabled faster identification of putative biomarkers [5], their validation is still hindered by low statistical power as well as limited reproducibility of results.

Herein, through meta-analysis we have evaluated the effect size of DR regimens on adipose mass and well-recognized biomarkers of healthy aging. Overall findings provide the geroprotective footprint of DR in humans and highlight a useful platform to validate or monitor the efficiency of dietary treatments to preserve and improve health span and longevity.

## RESULTS

### Effects of DR on total and visceral adipose mass

**Figure 1:**
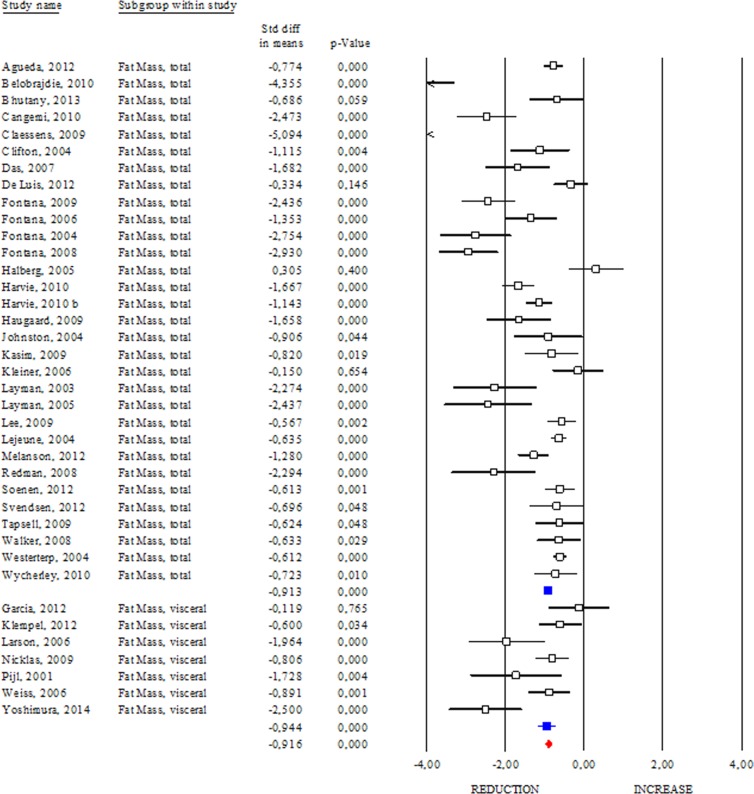
Changes of total and visceral adipose mass after DR Studies were stratified according to the design of the study. A positive standardized difference in mean (SDM) indicates an increase, whereas a negative SDM indicates the decrease of fat mass (total or visceral). The empty black square indicates the results of each study, whereas empty blu square shows the summary results of each subgroup data. The red diamond resumes overall results of the included studies in the forest plot.

DR regimens are effective in slowing aging, and maintaining healthy status in animals [6, 7]. Adipose mass quickly and dynamically responds to nutrient/energy fluctuation and its remodelling seems to mediate the beneficial effects of DR [7]. In this section we evaluated the effects of DR on adipose mass (Fig. 1). Interestingly, all studies showed clear evidence on the efficacy of DR in reducing total adipose mass in human (SDM −0.913; 95% CI −0.994, −0.832; p<0.000). Interestingly, we detected higher effectiveness of DR in healthy than unhealthy subjects (SDM −1.843; 95% CI −2.144, −1.542 p<0.000 and SDM −0.813; 95% CI −0.897, −0.728 p<0.000, respectively). Our data reveal that DR was also effective in reducing visceral fat mass (SDM −0.944; 95% CI −1.187, −0.700; p<0.000) (Fig. 1) and identify adipose mass measurement as a feasible approach to evaluate the efficacy of diets low in calories.

### Effects of DR on adipokines and DHEA

**Figure 2:**
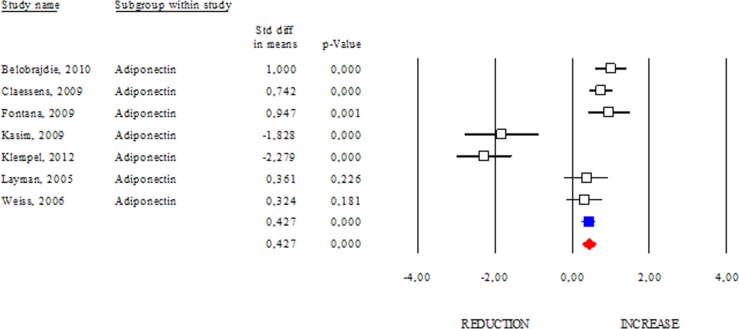
DR effects on circulating adiponectin Studies were stratified according to the design of the study. A positive standardized difference in mean (SDM) indicates an increase, whereas a negative SDM indicates the decrease of circulating adiponectin. The empty black square indicates the results of each study, whereas empty blu square shows the summary results of each subgroup data. The red diamond resumes overall results of the included studies in the forest plot.

**Figure 3:**
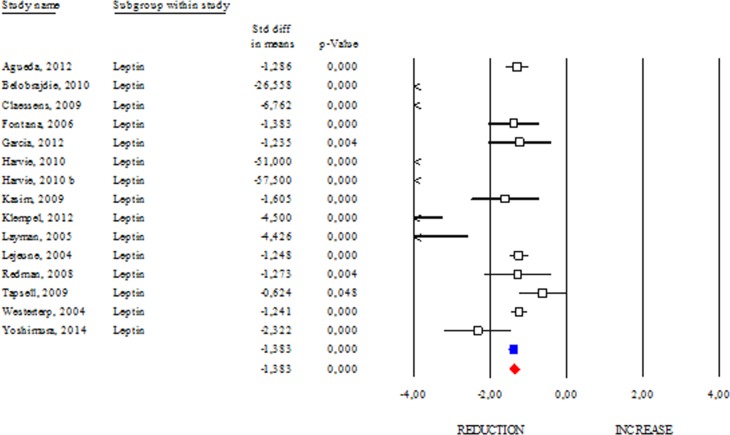
DR effects on circulating leptin Studies were stratified according to the design of the study. A positive standardized difference in mean (SDM) indicates an increase, whereas a negative SDM indicates the decrease of circulating leptin. The empty black square indicates the results of each study. The red diamond resumes overall results of the included studies in the forest plot.

Among adipokines, adiponectin has an anti-inflammatory function and correlates with healthy metabolic profile. Reduction of adiponectin production is often revealed in obese and diabetic subjects [8]. These evidences highlight adiponectin as a good candidate to monitor healthy status in human. However, conflicting results emerge from circulating adiponectin levels in centenarians [9, 10]. Herein we determined changes of adiponectin levels occurring after DR. As shown in Fig. 2, DR increased adiponectin levels in human (SDM 0.427; 95% CI 0.243, 0.612; p<0.000) independently of healthy status (healthy group: SDM 0.947; 95% CI 0.395, 1.499 p<0.001 and unhealthy group: SDM 0.370; 95% CI 0.155, 0.585 p<0.001). The “satiety hormone” leptin controls dietary behaviour and has been strongly associated with adipose mass. Indeed, reduced leptin levels are associated with diminished visceral adipose mass. However, unclear are evidences about its levels in healthy centenarians [9, 10]. Our data reveal that leptin levels were significantly reduced in DR group (SDM −1.383; 95% CI −1.511, −1.255; P<0.000) (Fig. 3).

The hormonal profile of aging includes a marked decrease in the adrenal hormone dehydroepiandro-sterone (DHEA) [11]. DHEA is taken up by adipose tissue and seems to reduce its mass protecting against obesity [12]. Epidemiologic data in the elderly cohort of long-living Okinawans (over 65) show relatively high plasma DHEA levels at older ages than the aged-matched counterpart [13]. However, as disclosed in Suppl. Fig. 1, DHEA levels were unchanged after DR (SDM 0.149; 95% CI −0.342, 0.641 p 0.551). Overall findings suggest a tight relationship between changes in circulating adipokines and reduction of adipose mass occurring after DR. Differently, DHEA modulation seems to be independent of calorie intake.

### Effects of DR on insulin, IGF-1, HOMA Index and IGBPs

**Figure 4:**
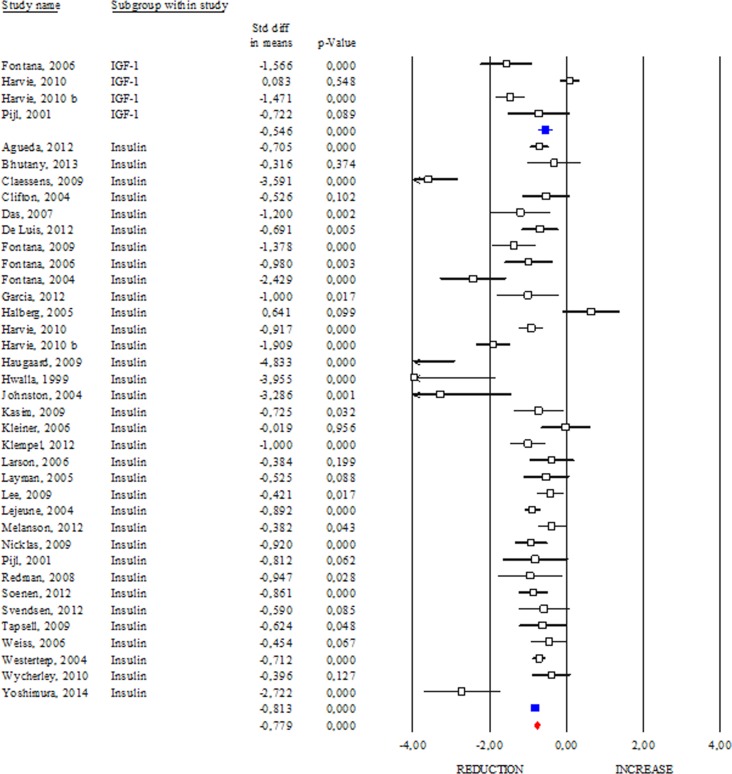
Changes of circulating insulin and insulin growth factor-1 (IGF-1) after DR Studies were stratified according to the design of the study. A positive standardized difference in mean (SDM) indicates an increase, whereas a negative SDM indicates the decrease of circulating IGF-1 or insulin. The empty black square indicates the results of each study, whereas empty blu square shows the summary results of each subgroup data. The red diamond resumes overall results of the included studies in the forest plot.

Insulin and insulin growth factors 1 (IGF-1) signalling is an evolutionary conserved pathway linking nutrient levels to fat mass and lifespan. Generally, reduced level of insulin and IGF-1 is associated with increased longevity from yeasts to mammals [14]. Differently, levels of insulin and IGF-1 are commonly higher in subjects affected by age-related diseases or obesity than lean healthy subjects [15]. In our work, we reported clear evidence about DR effects on insulin and IGF-1 levels in human (Fig. 4). In particular, we observed a significant reduction in insulin both in healthy (SDM −1.019; 95% CI −1.362, −0.675 p<0.000) and unhealthy subjects (SDM −0.811; 95% CI −0.893, −0.730 p<0.000). The same trend was detected by analysing the IGF-1 levels (SDM −0.546; 95% CI −0.750, −0.342 p<0.000). Overall data analyses (SDM −0.779; 95% CI −0.851, −0.706 p<0.000) confirm decreased insulin/IGF-1 levels as downstream effect of DR in human.

**Figure 5:**
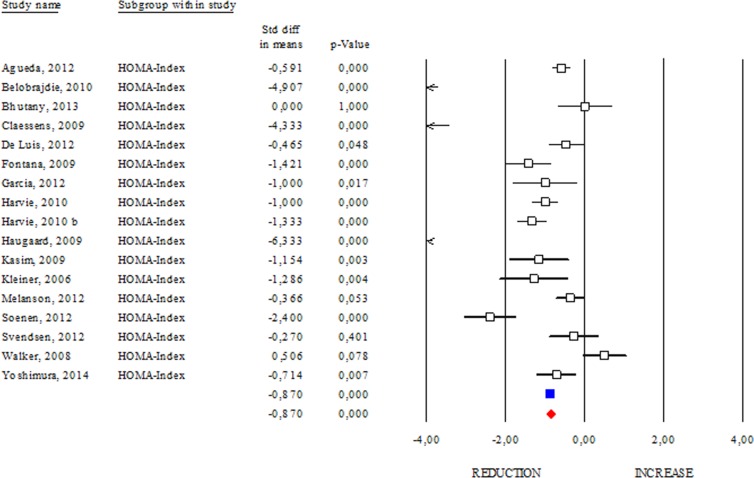
Changes of HOMA Index after DR Studies were stratified according to the design of the study. A positive standardized difference in mean (SDM) indicates an increase, whereas a negative SDM indicates the decrease of HOMA Index. The empty black square indicates the results of each study. The red diamond resumes overall results of the included studies in the forest plot.

The Homeostasis Model Assessment (HOMA) Index is currently a biochemical tool to estimate insulin sensitivity by matching fasting glycaemia and insulinemia [16]. A study carried out on centenarians indicates that they seem to be protected from hyper-insulinaemia, and their insulin resistance is as low, if not lower, than that of healthy younger adults [17]. The correlation between HOMA Index with obesity or aging suggests its prognostic capacity to evaluate the efficacy of health promoting strategies. Accordingly, we reported a significant reduction in the HOMA Index occurring after DR (SDM −0.837; 95% CI −0.990, −0.750 p<0.000) (Fig. 5) and this effect was stronger if dietary treatment was longer than 3 months (data not shown).

**Figure 6:**
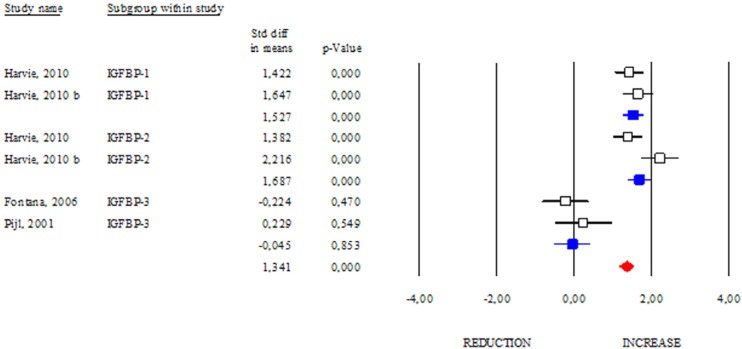
Changes of circulating IGFB-1, IGFBP-2 and IGFBP-3 after DR Studies were stratified according to the design of the study. A standardized difference in mean (SDM) indicates an increase, whereas a negative SDM indicates the decrease of IGFB-1, IGFBP-2 or IGFBP-3. The empty black square indicates the results of each study, whereas empty blu square shows the summary results of each subgroup data. The red diamond resumes overall results of the included studies in the forest plot.

The IGF-binding protein 2 (IGFBP2) is known as a carrier protein for IGF-1 limiting its biological action [18]. However, there are several characterized IGFBPs, which seem to improve metabolic status independently of IGFs binding [19]. Interestingly, some papers reported that DR regimens increase circulating levels of IGFBPs [20]. In our work, we analysed the changes in the levels of the best-known IGFBPs after DR. As shown in Fig. 6, DR similarly modulated IGFBP-1 and IGFBP-2 levels (SDM 1.527; 95% CI 1.248, 1.806 p<0.000 and SDM 1.687; 95% CI 1.387, 1.986 p<0.000, respectively). Differently, DR was ineffective in increasing IGFBP-3 levels (SDM −0.045; 95% CI −0.517, 0.427 p=0.853). These results suggest that IGFBP-1 and -2 are more sensitive to DR than IGFBP-3.

### Effects of DR on inflammatory markers

**Figure 7:**
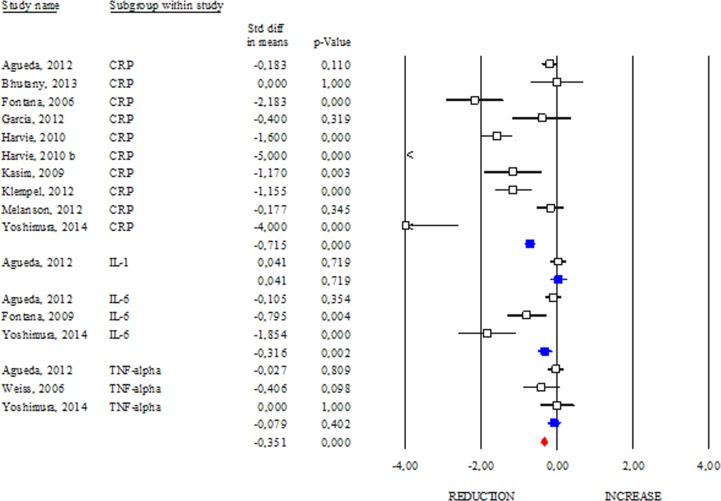
Changes of inflammatory markers after DR Studies were stratified according to the design of the study. A positive standardized difference in mean (SDM) indicates an increase, whereas a negative SDM indicates the decrease of CRP, IL-1, IL-6 or TNF-alpha. The empty black square indicates the results of each study, whereas empty blu square shows the summary results of each subgroup data. The red diamond resumes overall results of the included studies in the forest plot.

One of the common features of aging and obesity is the presence of a chronic sterile low-grade inflammatory status, which contributes to the onset of several metabolic perturbations [21]. In our work we evaluated the changes in circulating inflammatory markers observed after DR (Fig. 7). Interestingly, among the evaluated inflammatory markers, only CRP and IL-6 displayed a significant reduction after DR (SDM −0.715; 95% CI −0.862, −0.568 p<0.000 and SDM −0.316; 95% CI −0.515, −0.118 p<0.002, respectively). Although IL-1 and TNF-α are cytokines routinely assayed to monitor systemic inflammation, our data revealed that their level remained unchanged after DR (SDM 0.041; 95% CI −0.181, 0.263 p=0.719 and SDM −0.079; 95% CI −0.264, 0.106 p=0.402, respectively). Overall data regarding CRP, IL-6, IL-1 and TNF-α levels revealed anti-inflammatory effect of DR in human (SDM −0.351; 95% CI −0.442, −0.260 p<0.000) (Fig. 7).

## DISCUSSION

Aging is commonly defined as a physiological decline of biological functions in the body. Aging strongly remodels adipose depots by reducing subcutaneous adipose in favour of visceral depots enlargement [22]. Aging and visceral adipose tissue expansion act in synergy in inducing a chronic low grade of inflammatory status, which triggers a systemic metabolic decline in human [21, 23]. DR is a promising and feasible strategy that ameliorates body metabolic and inflammatory profile increasing lifespan through evolutionary-conserved mechanisms [4, 22, 24, 25]. Herein we included all studies evaluating the impact of DR on several healthy-associated markers in human including adipose mass. Increased visceral adiposity leads to chronic inflammation, which is often associated with a number of comorbidities (e.g. hyperinsulinemia, hypertension, insulin resistance, glucose intolerance) and reduced life expectancy [26, 27]. Through this meta-analysis approach, we confirmed the capacity of DR to reduce total and visceral adipose mass and, interestingly, we observed a more effective visceral adipose mass reduction after DR regimens (−20% in DR: SDM −1.081; 95% CI −1.242, −0.921 p<0.000) (−30/40% in DR: SDM −0.893; 95% CI −1.050, −0.737 p<0.000 and >-40% in DR: SDM −0.678; 95% CI −0.800, −0.555 p<0.000). These findings suggest that to obtain a more effective adipose mass loss, 20% in calorie reduction could be an elective strategy. Central or visceral adiposity perturbs systemic inflammation in animal models and human and relatively to this, the healthy effects of DR could be mediated by visceral adiposity reduction. Indeed, DR significantly diminished the markers of inflammation, highlighting the central role of DR-mediated adipose tissue remodelling in improving inflammatory profile in human. Furthermore, DR also increased adiponectin/leptin ratio, which is commonly associated with ameliorated insulin sensitivity in human. In line with this effect, we demonstrated that DR was successful in reducing insulin, IGF-1 and HOMA index.

The insulin growth factor binding proteins (IGFBPs) are a family of proteins that bind to insulin-like growth factors limiting their biological actions [28]. IGFBP-2 is the most abundant among circulating IGFBPs and its anti-diabetic role as well as direct ability to limit adipogenesis has been demonstrated [29, 30]. Actually, high serum levels of IGFBP-2 appear to protect against obesity and type 2 diabetes [30]. IGFBP-1 showed an inverse relation with insulin and BMI in human [31]. Differently, unclear are the evidences about the link between IGFBP-3 and adipose mass. In accordance with the data described above, we observed a strong responsiveness in circulating levels of IGFBP-1 and -2 occurring after DR. However some limitations emerge from this meta-analysis. In particular, statistical analyses on IL-1 and IGFBPs were carried out only evaluating the results obtained from few studies [32-35]. Moreover, it was not possible to evaluate the efficiency of DR in gender or time of treatment subgroups because it was difficult to collect a good number of subjects.

**Figure 8:**
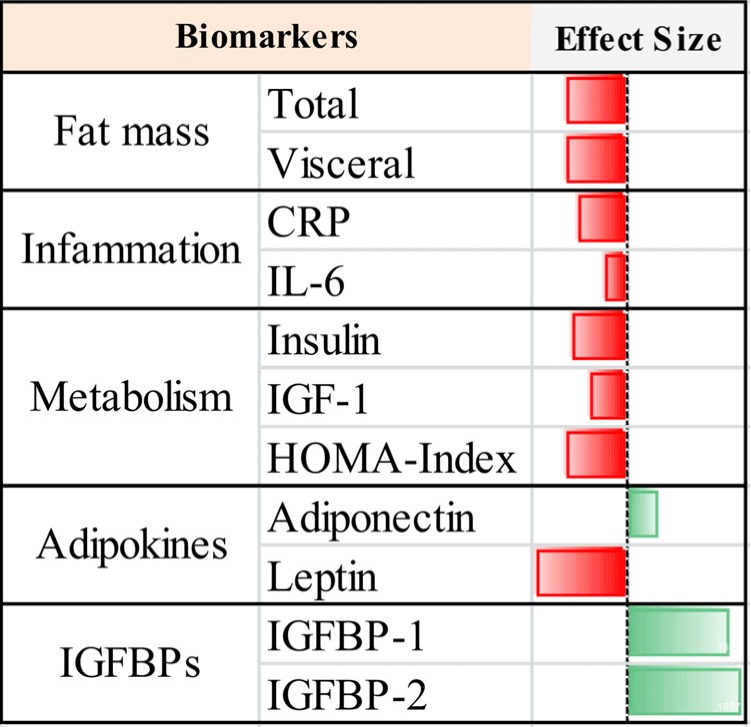
Geroprotective footprint of dietary restriction

**Figure 9:**
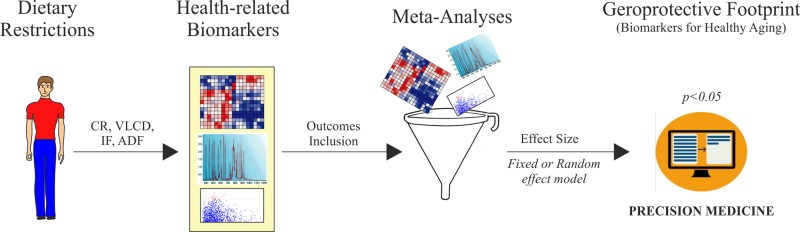
Algorithm development for biomarkers validation of dietary restriction in human CR: calorie restriction; VLCD: very low calorie diet; IF: intermittent fasting; ADF: alternate-day-fasting.

In conclusion, by a meta-analysis approach we have provided evidences about DR efficiency on key hallmarks of aging (Fig. 8) and built a useful platform to evaluate the responses of human to dietary regimens low in calories (Fig. 9).

## MATERIALS AND METHODS

### Search strategy and included studies

**Figure 10:**
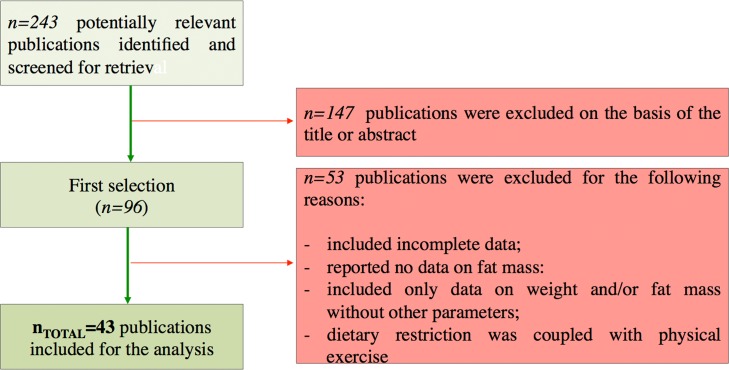
Flow chart of the study identification and selection

In our work we analysed human intervention studies and evaluated the impact of DR regimens on adipose mass and some biomarkers of healthy aging (*Geromarkers*). The *Geromarkers* included in our meta-analysis were described in Table 1. Two investigators, E.G. and D.L.B., independently carried out study selection and included both studies with an experimental design (EXP) and quasi-experimental design (Q-EXP). EXP studies were randomized with a control group and a parallel or crossover design; whereas Q-EXP included observational studies (pre- and post-intervention or pre- and post-data), non-randomized or uncontrolled studies [36]. Q-EXP studies were pooled together with EXP studies only after assessing whether they were in agreement with EXP studies [37]. Candidate studies were searched in PubMed (finalized February 30, 2016) using the terms ‘calorie or caloric or dietary restriction’, ‘fasting or intermittent fasting or alternate day fasting and ‘adipose tissue or fat mass or fat tissue’’. Inclusion criteria were as follows: human intervention studies with long-term study design (> 3 months); healthy and unhealthy (e.g. dyslipidaemia, obesity, metabolic syndrome) subjects; numerically analysable information about results, study duration and calories reduced in the study. Studies were excluded when: only abstracts were available; duration time of the study was lesser than 3 months; data presentation was incomplete; information about the DR was incomplete. When necessary, efforts were made to contact investigators for clarification or additional data. This research strategy produced a total of 201 studies. Furthermore, a manual research of references from clinical studies and reviews identified 42 additional studies, for a total of 243 studies to be evaluated, 9 of which are reviews [38-46]. A first screening allowed discarding 147 articles whose titles or abstracts were evidently irrelevant to our aim. Of the remaining 96 studies, 53 were rejected whenever: they presented incomplete data; DR was coupled with physical exercise; there were no reported data on adipose mass; they only presented data on weight and fat mass without other parameters (Fig. 10). Therefore, from 243 initial candidates, the 43 studies available for a formal meta-analysis had the following charac-teristics: they were written in English; they had a period of intervention of at least two weeks; they were carried out exclusively on human subjects. Among the considered studies, 12 were on females [32, 34, 35, 47-55], 4 on males [56-59], and the rest mixed [60-62, 33, 63-85]. Moreover, 30 studies were intervention studies evaluating the efficacy of calorie restriction [33-35, 47-49, 51-53, 56, 58-60, 63-67, 69, 71-73, 75, 76, 79-82, 84, 85]; 4 were intervention studies evaluating the efficacy of intermittent fasting [50, 57, 61, 70]; 9 were intervention studies evaluating the efficacy of low or very low calorie diets [32, 54, 55, 62, 68, 74, 77, 78, 83]. The selected studies included human groups with different BMI. In particular, 10 were studies on obese [34, 35, 48, 50, 53, 54, 58, 61, 64, 68], 16 on overweight [51, 52, 57, 59, 62, 63, 65, 69, 71-73, 77-79, 82, 85], 12 on both obese and overweight [32, 47, 49, 55, 60, 62, 74, 75, 80, 81, 83, 84], 5 on both normal weight and over-weight [33, 66, 67, 70, 76]. Finally, the studies were on healthy subjects, with the exception of few articles in which subjects were affected by the following pathologies: chronic osteoarthritis [64]; metabolic syndrome [59]; hyperinsulinemia [58, 72], polycystic ovary syndrome [49], type 2 diabetes [84]. Hence, the meta-analysis was based on 43 studies and analysed a total of 2094 subjects. Before analyses, all studies were stratified for gender, healthy status, time of treatment and percentage of calorie reduction and the main characteristics of the included studies were reported in Table 2. Calorie restriction, intermittent or alternate-day-fasting and low calorie diet interventions were overall grouped in dietary restriction (DR) category.

**Table 1:** Selected biomarkers and number of the studies included in meta-analysis

Biomarkers	n. of the studies
Fat Mass (total and visceral)	38
Adipokines (adiponectin and leptin)	22
IGFBPs (IGFBP-1, -2, -3)	6
IGF-1	4
HOMA-Index	17
Insulin	34
Inflammation (TNFa, IL-1, IL-6, CRP)	17
DHEA	5

**Table 2:** Characteristics of the included studies for the meta-analyses

Study Design				Gender Stratification		Healthy Status Stratification		Time of Treatment	
Unrandomized	Randomized or Controlled	Randomized and Controlled	Cross-Sectional	Yes	No	Yes	No	Brief (<3 months)	Long-Term (>3 months)
									
**4**	**14**	**23**	**2**	**16**	**27**	**36**	**2**	**26**	**16**

### Data analysis

Relevant data of the 43 studies available were entered for formal meta-analytic evaluation into the Comprehensive Meta-Analysis software (Biostat) [86]. Data analysis was performed as previously described [87]. In particular, for the results showed as post-data only, we selected mean, standard deviation and sample size in each group, or difference in means, sample size and p value between groups. When results were reported as pre- and post-data, we used mean, standard deviation, sample size in each group and correlation between baseline and end-point intervention period, or mean change, standard deviation difference, sample size in each group, correlation between baseline and end-point intervention period. For observational studies considering only one group (pre–post-intervention data), we used mean difference, standard deviation of difference and sample size. In all studies, we assumed the correlation between baseline and end-point study period to be 0.5 to produce the most conservative estimate [37, 88], To enable a joint comparison, the standardized difference in mean (SDM) was calculated for each outcome. In our analysis, positive SDM indicates increased effect size of DR on outcome considered. The effect sizes of the included studies were pooled both under a ‘fixed effects model’ or ‘random effects model’. Under fixed effects model we assumed that the true effect is the same in all studies. By contrast, under the random effects model we allowed that the true effect may vary from one study to the next [37]. Fixed or random effect model was selected following evaluation of heterogeneity between studies based on the *I*^2^ test for heterogeneity. When *I*^2^ values were low, we selected a fixed effects model, whereas random effects model was selected for *I*^2^ values higher than 75%.
